# Chemical Characterization and Enzymatic Control of Stickies in Kraft Paper Production

**DOI:** 10.3390/polym12010245

**Published:** 2020-01-20

**Authors:** Lourdes Ballinas-Casarrubias, Guillermo González-Sánchez, Salvador Eguiarte-Franco, Tania Siqueiros-Cendón, Sergio Flores-Gallardo, Eduardo Duarte Villa, Miguel de Dios Hernandez, Beatriz Rocha-Gutiérrez, Quintín Rascón-Cruz

**Affiliations:** 1 Facultad de Ciencias Químicas, Universidad Autónoma de Chihuahua, Circuito Universitario s/n, Campus Universitario No. 2, Chihuahua C.P. 31125, Mexico; mballinas@uach.mx (L.B.-C.); seguiarte@hotmail.com (S.E.-F.); tsiqueiros@uach.mx (T.S.-C.); brocha@uach.mx (B.R.-G.); 2 Centro de Investigación en Materiales Avanzados, S.C. Laboratorio Nacional de Nanotecnología, Miguel de Cervantes No. 120, Chihuahua C.P. 31109, Mexico; guillermo.gonzalez@cimav.edu.mx (G.G.-S.); sergio.flores@cimav.edu.mx (S.F.-G.); 3 Grupo COPAMEX, Ave. de la Juventud no. 280, San Nicolás de los Garza C.P. 66450, Mexico; eduardo.duarte@copamex.com (E.D.V.); miguel.dedios@copamex.com (M.d.D.H.)

**Keywords:** stickies, recycling, wastepaper, lipases, kraft paper

## Abstract

Paper recycling has increased in recent years. A principal consequence of this process is the problem of addressing some polymeric components known as stickies. A deep characterization of stickies sampled over one year in a recycled paper industry in México was performed. Based on their chemical structure, an enzymatic assay was performed using lipases. Compounds found in stickies by Fourier-transform infrared spectrometry were poly (butyl-acrylate), dioctyl phthalate, poly (vinyl-acetate), and poly (vinyl-acrylate). Pulp with 4% (*w/w*) consistency and pH = 6.2 was sampled directly from the mill once macrostickies were removed. Stickies were quantified by counting the tacky macrostructures in the liquid fraction of the pulp using a Neubauer chamber before the paper was made, and they were analyzed with rhodamine dye and a UV lamp. Of the two commercial enzymes evaluated, the best treatment condition used Lipase 30 G (Specialty Enzymes & Biotechnologies Co^®^, Chino, CA, USA) at a concentration of 0.44 g/L, which decreased 35.59% of stickies. SebOil DG (Specialty Enzymes & Biotechnologies^®^) showed a stickies reduction of 21.5% when used at a concentration of 0.33 g/L. Stickies in kraft paper processes were actively controlled by the action of lipases, and future research should focus on how this enzyme recognizes its substrate and should apply synthetic biology to improve lipase specificity.

## 1. Introduction

Secondary fibers are the principal feedstock for paper production worldwide, principally due to environmental and economic issues, regarding the use of recycled materials rather than pure cellulose. It was reported that 40% of total paper production is based on the use of such fibers. A principal consequence of fiber recycling is the need to deal with some contaminants known as stickies [1]. These compounds are provided by the raw materials used in the manufacture of paper, and their appearance is enhanced by closed mill water loops. Stickies come from inks and special adhesives principally used in recycled fibers such as OCC (old corrugated containers), MOW (mixed office waste), and ONP (newsprint); they may be presented in pulp, process water, or the final product [2]. The stickies term does not imply any particular chemical composition but is derived from the complex physical and chemical nature of a mixture of organic substances, which are tacky, hydrophobic, and pliable, with variable shapes, and which are denser than water. Stickies can deform and aggregate with temperature changes, have a wide range of melting points, and act as adhesives [3]. Stickies can be classified based on their size and tendency to agglomerate [4]. Macrostickies are larger and cannot pass through a 100-micron-slotted screen plate. They are associated with the fibrous fraction of pulp. Microstickies range in dimension from above 1 micron to below 100 microns and are related to the fine fraction of the pulp. The smaller fraction corresponds to colloidal stickies, which are smaller than 5 microns. These are more difficult to control because they can precipitate or lead to a change in physical state due to system imbalances, such as in pH or ionic strength [5,6]. Knowledge of their properties is helpful to address problems related to stickies.

The principal concerns related to stickies are deposits within the paper machine and on the paper itself [3]. The first type of deposit occurs in the wires, felts, and rolls and produces lengthy, non-productive cleaning times. Deposition also produces paper brakes, where spots adhere to the moving rolls. Deposition into the paper reduces its quality and creates stacking problems between sheets [7,8].

Stickies can be minimized using chemical and mechanical methods. The former are mainly used for the smaller fraction, and the latter are used for macrostickies. Mechanical methods principally use the following operations: Screening, dissolved air flotation, and washing with water. These can be used in combination with chemical methods to optimize the cleaning procedure [6]. With mechanical procedures, only the bigger stickies are removed. Chemical procedures are the principal method for control of micro and colloidal stickies. This control is based on the use of dispersants, adsorbents, and diverse additives [4], whose chemical properties are detrimental for their specific action on the identified stickies. Most methods passivate the surfaces of stickies via pH and temperature adjustment, and thus, the surfaces are cleaned, avoiding deposition of stickies. Nevertheless, the problems caused by the appearance of stickies are not easily handled, and there is no single effective method to manage them. Stickies control is judged based on paper quality and the reduction in cleaning procedures; thus, another important issue is to quantify stickies and standardize their measurement [8].

Various methods are available for the quantification of stickies and can be classified into methods that measure the quantity, composition, or deposition tendency [8,9]. There are also morphological determinations using screening and microscopic analysis, which can be helpful for macrostickies. In the case of colloidal and microstickies, chemical analysis based on solvent extraction procedures and gravimetric analysis can be used. There are also reports on thermogravimetric analysis (TGA), Fourier-transform infrared spectrometry (FTIR), and nuclear magnetic resonance spectrometry (NMR) studies [10]. When the water fraction is evaluated, it is possible to use a fluorescent counting method [6], or turbidimetry. However, there is no reliable and repeatable method that is accepted as standard.

There are two main priorities for the management of stickies during the papermaking process, i.e., elimination and prevention of adhesions, and removal of as many stickies as possible [9]. As described above, the presence of stickies leads to loss of production time and increased production costs; since 2010, the Confederation of European Paper (CEPI) reported an annual cost of $700 million. Whereas, Monte et al. [10] and CEPI [11] estimated losses of up to $218 million a year due to the use of recycled fiber; Bajpai [4] in 2012 reported a cost of $500 million dollars annually, in the USA alone.

At Copamex (Pachisa) Mexico, stickies control and production losses are as high as 4.5 million dollars/year, and thus, it is a priority for the company to improve stickies management. The present work is a complete study performed over one year of operation for a complete characterization of stickies. The sampling procedure was designed to determine a specific method for quantification of stickies or a strategy to minimize their impact during papermaking [6]. As mentioned, there are several methods for controlling stickies; the company has evaluated flotation/dispersion [12,13], mechanical methods such as a rotating wire-mesh-analyzer, and physical-chemical methods [2,14] using talc for passivation of stickies. In this study, given the growing environmental concerns around process innovation, a biotechnological route was implemented. The paper industry has been a pioneer in the use of enzymes in process methods [13,15]. Various enzymes (esterases) have been reported that have the ability to degrade components of stickies, preventing agglomeration and its consequences [16,17,18,19]. The number of studies for sticky control using lipases is very limited. Lipases, in addition to cellulases, were effective in removing anionic residues and pitch deposits in the whitewater from papermaking [20]. Pulping with either cellulase, lipase, or a mixture of both enzymes at the ambient pH of mixed office paper, removed the adhesives effectively [21]. Lipases have been used more frequently for pitch control. The lipophilic extractives originate pitch, which affects the entire pulp and paper manufacturing processes. White-rot fungi and their enzymes have been demonstrated to be effective in pulping traditional methods [22,23]. Enzymatic preparations for the control of triglyceride-containing pitch deposits during the manufacture of mechanical and sulfite paper are also commercially available [24,25]. In this study, the principal aim was to evaluate commercial lipases for stickies mitigation. These enzymes hydrolyze lipids, releasing fatty acids and glycerol. In addition to stickies reduction, the by-products produced could act as surfactants preventing future agglomeration of stickies. This work focuses on characterizing stickies directly sampled from the factory and studying the effects of two commercially available enzymes with lipase activity (SebOil DG and Lipase 30 G) on their reduction. It also applied a stickies quantification procedure that was as simple as possible, both during the paper-making process and to the water fraction, to evaluate the method’s effectiveness.

## 2. Materials and Methods

### 2.1. Stickies Sampling

Sampling was performed in August 2012 at Papelera de Chihuahua, S.A. (Chihuahua, Mexico). The temperature was measured, and polypropylene bottles were used for storage. Solid stickies were taken from the felts and coils of the paper machine, and the water fraction of the pulp was collected from the trays of the paper machine. The samples were used immediately or stored for no more than 1 h at 4 °C until use. 

Soxhlet extraction was performed with various organic solvents in an extraction train. First, the sample was shredded manually due to the plasticity of the components and placed in a vacuum desiccator. Thimbles for Soxhlet extraction were dried for 48 h and weighed until a constant mass was obtained. Stickies were placed into the thimbles and covered with glass fiber. The Soxhlet system was set as follows: 200 mL of each solvent was poured into a round-bottomed flask and heated with a heating mantle. The flask was connected to a Soxhlet extractor with the loaded thimble. A condenser was attached to the top of the system. The average contact time was 8 h. At the end of the procedure, the solvent was cooled and poured into an evaporator (Switzerland Buchi, Flawil, Switzerland) to concentrate the sample. The extract was refrigerated for further analysis.

The samples were identified by sampling month and the collection location. The first number corresponds to the sampling month, and the second and third correspond to the origin, as follows:

- B3—Coil 3;
- B4—Coil 4;
- LS—Dirty felt.

The fourth and fifth letters indicate the nature of the sample and extract:

- M—Original sticky, product sampling;
- E—Extracted with ethanol;
- PE—Precipitated from ethanol extract;
- EA—Extracted with ethyl acetate;
- CH—Extracted with cyclohexane;
- DM—Extracted with dichloromethane;
- D—Sticky remaining in the Soxhlet thimble at the end of the entire extraction.

Table 1 shows the physicochemical characteristics of the organic solvents used. The hierarchy was established based on the magnitude of the orientation polarization, which includes both electronic and atomic polarization. The Hildebrand solubility parameter, which is used to predict the interactions of an organic solvent with a particular organic polymer, was also used.

### 2.2. Fourier-Transform Infrared Spectrometry

FTIR spectroscopy was performed using a Perkin Elmer (Waltham, MA, USA) (PE) FTIR spectrometer in the ATR (attenuated total reflectance) mode. It was equipped with a deuterated triglycine sulfate (DTGS) detector and a potassium bromide (KBr) beam splitter. A small amount (200 µL) of extract was dispersed and placed on the ATR cell. A total of 25 cumulative scans were taken, with a resolution of 2 cm^−1^ in transmission mode.

### 2.3. Thermogravimetric Analysis

Thermogravimetric (TG) measurements were made on an STD Q500 TA Instrument (New Castle, DE, USA). Samples of approximately 10 ± 3 mg were placed into alumina pans. The samples were heated from 25 to 1000 °C at 5 °C/min under an inert atmosphere (argon 99.99%) flowing at 60 mL/min for 60 min. At least three replicates were performed for each sample. The microbalance had a precision of ±0.1 µg.

### 2.4. Enzyme Preparation and Sample Treatment

Commercially available lipase-enzymes 30 G and SebOil (kindly provided by Specialty Enzymes & Biotechnologies, Chino, CA, USA) with triacylglycerol hydrolase activity were used as agents for the removal of stickies. Commercial enzyme purity was analyzed by polyacrylamide gel electrophoresis [26] and prepared in a final concentration of 80 mg/mL of total protein. The commercial enzymes were added to the sample (cellulose from the pulping process at 90 *w*/*w*% consistency) at different concentrations (0, 0.11, 0.22, 0.33, and 0.44 g/L). To mimic retention time in the papermaking process, enzymes were put into contact with the sample for 2 h under constant agitation at 37 °C. Stickies were quantified in triplicate for each treatment.

### 2.5. Stickies Quantification to Evaluate Enzymatic Treatment

Stickies quantification was performed in the pulp using a counting method developed in the kraft paper factory (Pachisa, Chihuahua, Mexico). It is continuously used to monitor stickies in the aqueous phase (usually secondary). A Neubauer chamber was used to count the colloidal particles present in the liquid fraction of the cellulose sample. Each sample (50 mL) was centrifuged at 500 rpm for 15 min, and an aliquot of 200 μL of the liquid phase material was transferred with an automatic pipette to the Neubauer chamber. Particle concentration was determined according to the following relationship (Equation (1)):

Particle Concentration = (*A*·*K*·*D*)/*V* (1)

where *A* = average number of particles per square; *K* = constant correction of glass curvature (calculated as 1.1); *V* = volume of liquid present in a square of the Neubauer chamber (2.5 × 10^−7^ cm^3^); and D = dilution factor of the sample. 

Stickies were visualized with an optical microscope (Olympus BX-4, Tokyo, Japan) using a 40 × 10 objective and supplied with a camera attached to the ocular viewer (Hitachi KPD50u, Tokyo, Japan). Data were statistically analyzed by ANOVA (Minitab, State College, PA, USA). To compare the different treatments made, a Dunnett’s test was performed at a confidence level of 95%.

Stickies were also evaluated using the following method. After enzymatic treatment, 0.1 mL of rhodamine was added to 100 mL of pulp and was shaken for 5 min. Paper was made using 200 to 300 mL of pulp in a micro-paper machine in the factory, in quadruplicate for each treatment. Stickies were revealed using UV light (366 nm), which counted spots revealed by the colorant, and were also gravimetrically measured.

## 3. Results

### 3.1. Stickies Extraction, TGA, and FTIR

Solid stickies were obtained directly from the paper machine in order to elucidate their structure and chemical composition. For the samples taken, a solvent Soxhlet treatment was performed using ethanol as the most polar treatment, followed by ethyl acetate, cyclohexane, and dichloromethane. In Table 2, the weight fractions of the analyzed stickies are shown for each solvent tested. This evaluation includes important information regarding the possible composition of the stickies analyzed. In general, stickies from B4 had the highest solubility. For the B3 and B4 samples, the total extracted fraction was very similar, but with different composition. In the polarity data, ethanol is the solvent with the highest fraction extracted for the samples evaluated, followed by ethyl acetate for B3, and cyclohexane for B4. Dichloromethane does not dissolve an important fraction of compounds present in B3 and B4.

**Figure 1:**
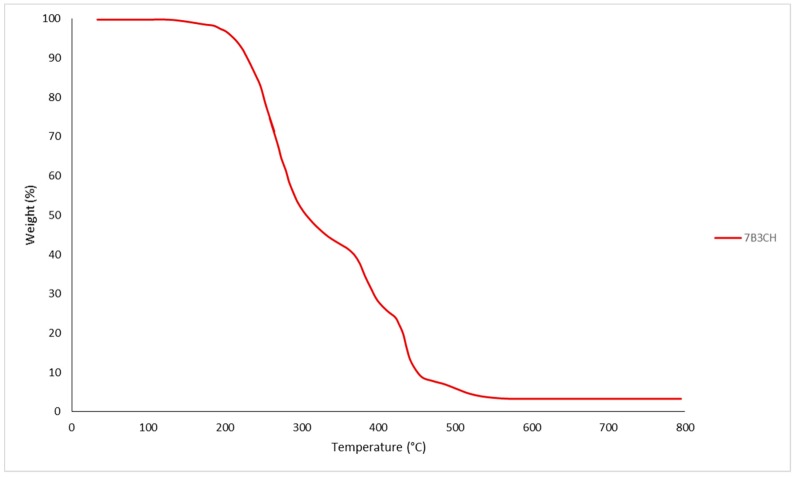
TGA analysis curve for stickies 7B3CH.

Thermogravimetric analysis is based on the weight loss produced by the pyrolysis process, measured as a function of temperature. The correlation of different weight fractions calculated from the solvent extraction and the TG data was obtained. From 200 to 500 °C ± 25 °C, the weight loss was attributed to organic compounds in the sample. Three different slope changes were observed and denoted as Comp A, B, and C, respectively, as shown in Figure 1.

The weight loss from 500 ± 25 °C to 600 °C is correlated to the total carbon in the sample, which is detached (fixed carbon). The sum total of the fractions and the fixed carbon is denoted as % weight of organic compounds. From 600 to 800 °C, a slight decrease is observed due to liberated CO_2_, which is produced by the following reaction:

(2) $${\mathrm{CaCO}}_{3}\to \mathrm{CaO}+{\mathrm{CO}}_{2}$$

If the molecular weight of CaCO_3_ is 100 g·gmol^−1^, and that of CO_2_ is 44 g·gmol^−1^ and of CaO is 56 g·gmol^−1^, the quantities of CaCO_3_ and CaO can be calculated from the CO_2_ liberated using the following relationships:

% CaCO_3_ = (100 g·gmol^−1^ × %TGA CO_2_)/44 g·gmol^−1^ (3)

% CaO = (56 g·gmol^−1^ × %TGA CO_2_)/44 g·gmol^−1^ (4)

The fraction remaining after 800 °C is composed of ashes containing silicon oxides, alumina silicates, titanium oxides, and others. In Table 3, the global results obtained by TG for the various extracts of B3 are shown. From the analysis of data, the following can be stated. The original sample (7B3M) contains three different organic fractions (Comp A, Comp B, and Comp C), and a relatively low fixed carbon quantity. The total organic fraction corresponds to 75% of the solid sample. After all solvent extractions, the percentages were as follows: 57.47% of the solid was not dissolved, 19.77% corresponds to ashes, and 21.90% was extracted. This balance corroborates the TG data. The inorganic fraction is composed mainly of calcium carbonate, which cannot be removed at the end of the solvent extraction procedure (see sample 7DB3). The fractions extracted with cyclohexane and dichloromethane reveal different transitions of the components.

**Figure 2:**
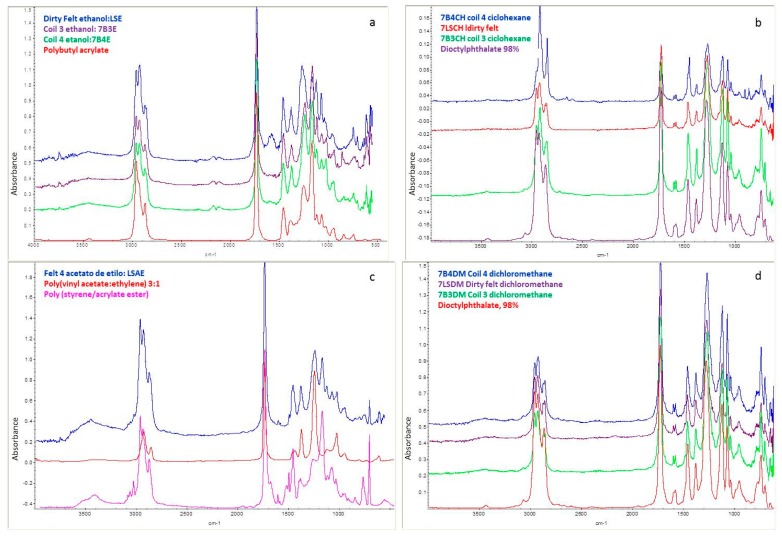
Comparison of the FTIR spectra of the different fractions of 7B3, 7B4, and 7LS. (**a**) Ethanol extraction; (**b**) cyclohexane; (**c**) ethyl acetate; (**d**) dichloromethane.

All fractions vary if they are compared to corresponding fractions in B3. The temperature ranges of the transitions are similar, but not the quantities present. There is little information about the TG analysis of stickies. The method is commonly used without extraction as a pretreatment. Weight loss between certain temperatures can be used to calculate the amount of stickies. The results are intrinsically related to the chemical nature evidenced by FTIR. Several FTIR spectra were acquired for the fractions described in the section on stickies extraction and TGA. The principal results are shown in Figure 2. A rough comparison of the entire spectra provides insight into the chemical nature of the compounds extracted by ethanol, ethyl acetate, dichloromethane, and cyclohexane. The principal observation is that the chemical nature of the compounds solubilized from the stickies sampled in B3, B4, and LS is the same. Although the structure and the macroscopic appearance of the stickies were different, the spectra almost overlap for each sample in each solvent tested, as shown in Figure 2. The first spectra (2a) are for the ethanol extracts of samples obtained in B3, B4, and LS. The spectra were analyzed directly with the database found in the PE spectrometer. For the ethanol fraction that was not solubilized, the main compound was poly (vinyl acetate/ethylene) 4:1 (data not shown). Vinyl acetate ethylene (VAE) emulsions are based on copolymerization of vinyl acetate (VA) and ethylene, with vinyl acetate content of 60–95% and ethylene content at 5–40%. This product differs from ethylene vinyl acetate (EVA) copolymers, in which vinyl acetate is 10–40% and ethylene is 60–90%.

Figure 2 shows the spectra of the different extracts. The correlation is related to dioctyl phthalate, which is one of the more common plasticizers used in the packing industry. In the spectra, the C-H stretching at 2999, 2933, and 2876 cm^−1^; the C=O stretching at 1726 cm^−1^; the C–O stretching of O–C=O at 1285 cm^−1^; the CO stretching of O–CH_2_ at 1077 cm^−1^; and the out-of-plane bending of the aromatic system at 745 cm^−1^ are observed. This compound predominates in the dichloromethane fraction.

The data obtained from the solubility, TG, and FTIR analyses indicate an extremely complex stickie mixture for each sample. The solvent with the highest polarity, Hildebrand parameter, and permittivity was ethanol, as shown in Table 1. In this fraction, one compound predominates, as revealed by TG in samples B3 and B4. The FTIR spectrum mainly corresponds to poly (butyl-acrylate) (PBA). When cyclohexane is employed, at least three organic fractions were evidenced by TG, but only one by FTIR—dioctyl phthalate. This solvent has the lowest polarity and Hildebrand parameter, and the stickie analyzed in TG should be an aggregate with other less concentrated components. Importantly, poly (vinyl acetate/ethylene) is one of the main compounds revealed in the non-soluble fraction for ethanol and soluble fraction in ethyl acetate.

**Figure 3:**
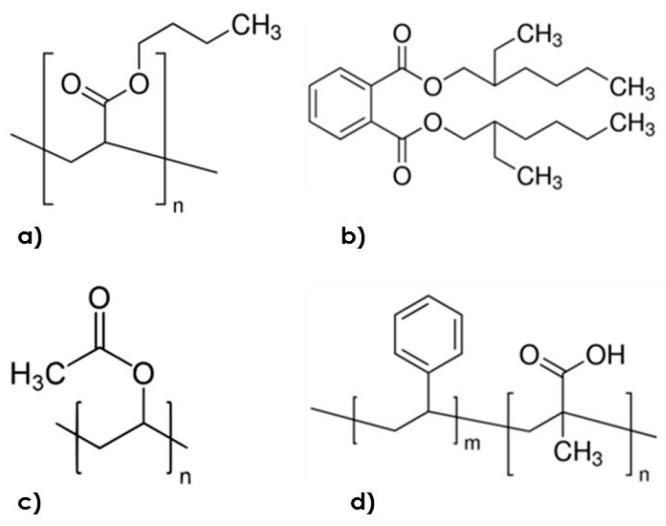
Compounds found in stickies by FTIR. (**a**) PBA; (**b**) dioctyl phthalate; (**c**) poly (vinyl-acetate); (**d**) poly (styrene-acrylate).

This study finds that the evaluated compounds forming the solid deposits in the paper machines of the factory are mainly carboxylic esters, as shown in Figure 3; these can be hydrolyzed by lipases, such as those evaluated in this work.

### 3.2. Determining the Purity and Activity of Lipase on Naturally Occurring Stickies

**Figure 4:**
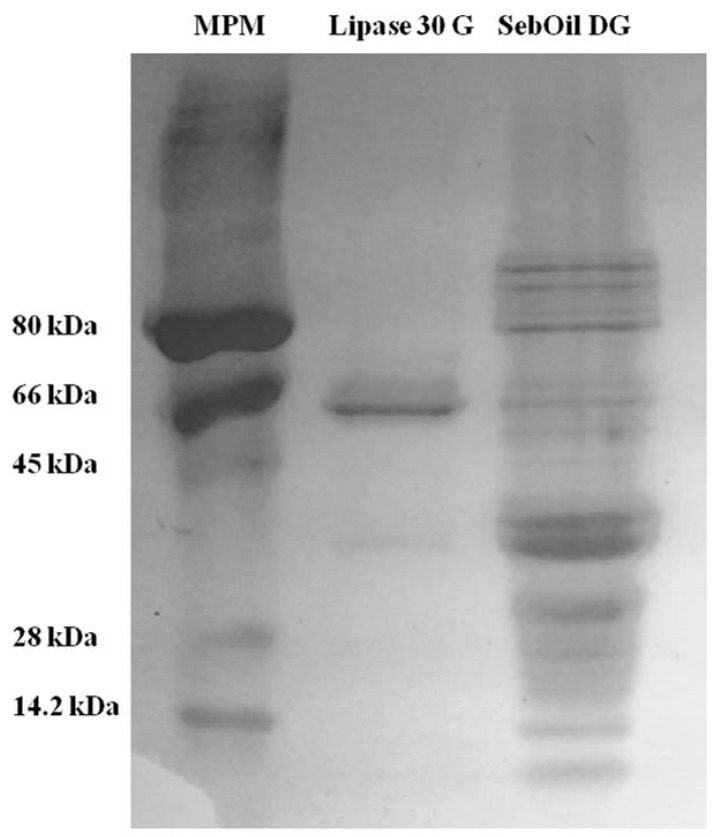
Molecular characteristics of the commercial enzymes (Lipase 30 G and SebOil DG) were determined by the electrophoretic technique.

The molecular characteristics of the commercial enzymes (Lipase 30 G and SebOil DG, Advanced Enzyme Technologies Ltd., Thane, India) were determined by electrophoretic techniques. Figure 4 shows the electrophoretic mobility of proteins in the Lipase 30 G and SebOil DG enzyme products that show lipase activity. According to this complexity, Lipase 30 G contains a well-defined protein of ~66 kDa and other two proteins at much less concentration. The same analysis for SebOil DG shows a group of high-molecular-weight proteins ranging from 60 to 120 kDa and a group of low-molecular-weight proteins ranging from 14.2 to 30 kDa. This analysis reveals the complexity of this formulation. 

**Figure 5:**
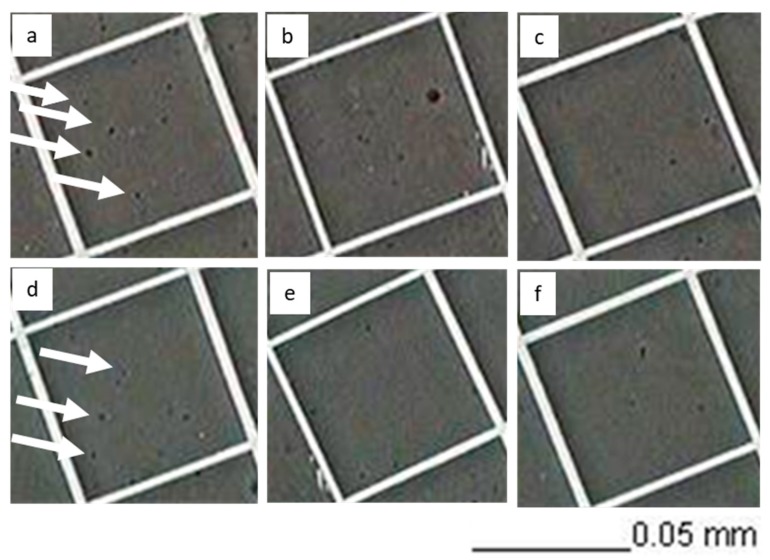
Effect of enzyme Lipase 30 G at different concentrations on the stickies. (**a**,**b**) control; (**c**) 0.11 g/L; (**d**) 0.22 g/L; (**e**) 0.33 g/L; and (**f**) 0.44 g/L. Large stikies are denoted by an arrow.

**Figure 6:**
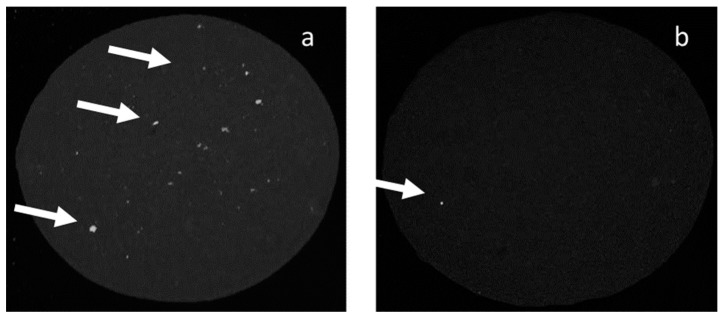
Stickies in paper using the rhodamine method. (**a**) No enzyme added; (**b**) treated with Lipase 30 G at 0.44 g/L. Large stikies are denoted by an arrow.

To determine the number of stickies in the samples and evaluate the action of enzymes in stickies reduction, a counting method developed in the factory was used. Various enzyme loads were tested and resulted in stickies removal, as shown in Table 4. Microscopic visualization of the enzymatic treatment is shown Figure 5. Both commercial enzymes could decrease the number of stickies present in the pulp. The observed activity of the enzyme Lipase 30 G increased as the enzyme concentration increased. The enzyme SebOil DG did not show the same behavior, but rather presented an activity maximum at a concentration of 0.33 g/L (data not shown). Stickies were also analyzed in the paper using rhodamine dye and a UV lamp as seen in Figure 6, and the refractive nature of the stickies was observed, as shown in Figure 6a. The data in Table 4 correspond to the activities attained with the enzyme Lipase 30 G. From the four treatments used, three showed a significant difference from the control (an ANOVA and Dunnett’s test, at a confidence level of 95%, were performed). The major removal efficiency (35.59%) was achieved at a concentration of 0.44 g/L, but statistically, all concentrations, 0.44, 0.33, or 0.22 g/L, reduced stickies. At a concentration of 0.11 g/L of enzyme extract, there was also a reduction in contaminants, but it was not significantly different from the control. Data in Table 4 show the enzymatic activity with SebOil DG. In this case, the four treatments used showed significant differences from the control. A major decrease in stickies (21.5%) was achieved at an enzyme concentration of 0.33 g/L, but a concentration of 0.11, 0.22, or 0.44 g/L of enzyme extract is expected to give a reduction in stickies. In Table 5, the results for stickies measurement in the formed paper are shown using 0.44 g/L of each enzyme. From these data, it can be concluded that Lipase 30 G strongly impacts the reduction of stickies associated with the produced paper. 

SebOil is a lipase that hydrolyzes the ester bond forming the corresponding alcohol and carboxylic acid. It can be used at 30–35 °C and pH = 5–6. Lipase is a triacylglycerol lipase that hydrolyzes any position in fatty acids. It can be used up to 45 °C and at pH = 5–8. Both enzymes operate at a temperature that is not the optimum reported, but with a good effectiveness in the aqueous fraction and in paper formation.

## 4. Discussion

Stickies cause many operational problems due to their complexity and variability in paper recycling processes. They are present as soluble (secondary stickies) and insoluble (primary stickies) components under normal conditions and dramatically change their behavior by inducing changes in wet-end chemistry, temperature, or pH [9]. Some mechanical processes remove larger stickies, but nevertheless, original microstickies (colloidal fraction) can agglomerate when the temperature rises to form larger tacky structures that deposit onto paper and paper machine structures. Secondary stickies originate from dissolved and colloidal compounds that cause deposit problems in paper machines. Authors, such as Doshi et al. [8] and Derakhshanian and Banerjee [27], have reported that stickies contain fibers, even though the association is weak. The same authors report that fillers and stickies can exist as agglomerates, such as talc, calcium carbonate, and kaolin, which are commonly used to detackify stickies.

Solvent extraction is commonly used to separate hydrophobic substances from pulp or deposit samples. Dichloromethane is the most used solvent for stickies extraction [28]; this solvent is widely used in adhesive solutions. Other authors have measured the solvent effectiveness by calculating a gravimetric value. Cao and Heise [29] calculated this value for a comparison, extracting wax from OCC. Hexane and acetone gave similar gravimetric values related to the amount of organic compounds extracted in mass. They found no significant difference among the acquired FTIR spectra and reported ethanol-benzene as a superior solvent in terms of the calculated gravimetric value. Different authors reported sequential extraction with various solvents as the best alternative to overcome solubility limitations for the complexity found in stickies samples, but this process is intensive and time consuming [30].

There are little data on TG analysis of stickies; residues are commonly extracted without pretreatment. The weight loss variance with temperature could be used to measure the availability of stickies. However, fiber material is partially pyrolyzed at the same temperature as stickies; the long fibers must be screened out before measurements [31]. To our knowledge, this is the first report of TGA for different extraction fractions collected from stickies samples. 

Fourier-transform infrared spectrometry is used to identify functional groups in organic compounds. This technique has been reported in the identification of stickies [32]. VAE are used for paints and coatings, as well as water-based adhesives for paper packaging [33]. FTIR spectra exhibit strong acetate ester carbonyl bands at 1737 cm^−1^ and an ester C–O stretch band at 1236 cm^−1^ arising from polyvinyl acetate. The characteristic absorbance bands are located at 2921, 2852, 1467, and 720 cm^−1^. VA predictions are calculated from the calibrated VA FTIR method for validation standards, at 0.55% VA and 1.00% vinyl acetate in polyethylene [34]. In the case of the ethanol fraction, the spectra are correlated with PBA. The C=O group stretching of PBA is at 1737 cm^−1^, and the characteristic absorption bands of the C–H stretching vibration and the C–H in plane-bending vibration appeared at 2876, 2930, 1380, and 1470 cm^−1^ [35]. The extracts prepared with ethyl acetate correlate well with the overlapping of poly (styrene-acrylate) spectra and poly (vinyl acetate/ethylene). For poly (styrene-acrylate) ester, the FTIR shows C–H bands of the benzene ring at 3010, 1600, and 1475 cm^−1^, and at 1712 cm^−1^ for the C=O band of acrylic acid. The literature contains some information on stickies composition. Johansson et al. [32] reported significant amounts of poly (vinyl-acetate), acrylates, and butadiene. Sjarfrom et al. [36] found EVA, SBS (styrene-butadiene-styrene), and acrylic polymers in wire deposits. 

The electrophoresis analysis reveals the complexity of commercial enzyme formulations. It is common to use complex protein mixtures or crude extracts derived from microorganisms to control pitch problems [16,37,38]. However, to develop feasible stickies control, it is important to study each microorganism-derived protein [39,40] or improve the over-expression of tailor-made recombinant proteins [41,42,43]. Another product consisting of esterase enzymes has been developed by Buckman and is known as Optimyze. This product breaks down stickies into smaller compounds. The conditions for operation are pH between 6.5 and 10; temperature range from 25 to 60 °C, and contact time of 45 min [17]. They reduce larger stickies to zero, with a total reduction of 90% in measurable stickies. However, the authors used a measurement method very different from the one we followed. The screening and pressing were followed by a scanner to measure stickies. A Pulmac MasterScreen was used to collect stickies from pulp on a pad. The stickies were transferred to a transparency using a laminator and measured with a flatbed scanner. Barba Cedillo et al. [44] reported the use of lipase in recycled paper. They attained over 30% sticky removal. Several authors have reported a number of esterase-type enzyme mixtures, primarily to reduce the size of the sticky. Many case studies were described by Bajpai [4] in 2012 involving all grade sectors, where stickies were reduced dramatically by enzymatic control programs. For non-enzymatic treatments, addition of cationic talc has been shown to control stickies as measured by the aluminum foil method. A 63% sticky removal level was observed in deinked pulp with the addition of 2.0% cationic talc [14,28]. 

As mentioned, Lipase 30 G is a triacylglycerol lipase enzyme produced by controlled fermentation of *Candida rugosa*. Lipase 30 G hydrolyzes short, medium, and long fatty acids from the 1, 2, and 3 positions of triacylglycerol. It reduced stickies in aqueous fraction and also in paper effectively, as shown in Table 4 and Table 5. SebOil DG is a lipase that hydrolyzes the ester bond in carbon 2 of phospholipid molecules, then adds a hydroxyl group to carbon 2, which releases a free fatty acid molecule. The formation of a hydroxyl group on carbon 2 makes the phospholipid molecule soluble in water. The mixture was not as effective as Lipase 30 G.

These lipases showed specificity towards the ester group of stickies components, resulting in a mixture of polymer products. Further studies regarding the specificity and characterization of the commercial enzymes should be driven to understand the mechanism of action during stickies mitigation.

## 5. Conclusions

The present study shows the results obtained by a systematic sampling of stickies directly obtained from a paper machine located at Chihuahua, Mexico. Extraction methods by a sequence of solvents were made, and the different fractions were evaluated by FTIR and TGA. Total extracts were measured, and ethanol was the solvent which presented the highest solubility for stickies components. The principal compounds found were vinyl acrylates and styrene-butadiene, which were recognized by FTIR. During sampling, pulp at 90% of consistency, pH = 6.2 was taken once the macrostickies were removed, directly from the mill. Stickies were quantified by means of the counting of the tacky macrostructures in the liquid fraction of the pulp, using a Neubauer chamber before the paper was made. To corroborate their treatment, stickies were also analyzed in the paper using rhodamine dye and a UV lamp, and measured by a gravimetric method. For the evaluation during the paper making process, a Neubauer chamber count technique was used; in paper, a rhodamine UV light-count was used. The enzymatic method developed, based on the use of commercially available Lipase 30 G and SebOil, and based on the application at different concentrations (0, 0.11, 0.22, 0.33, and 0.44 g/L), attained stickies removal up to 70%. Of the two enzymes evaluated, the best treatment condition was using Lipase 30 G at a concentration of 0.44 g/L, which produced a 35.59% stickies decrement in the aqueous fraction. SebOil DG presented a lesser stickies reduction of 21.5% when it was used at a concentration of 0.33 g/L. Stickies were actively controlled in kraft paper processes by the action of lipases.

## Figures and Tables

**Table 1:** Organic solvents used, *n*: Refractive index; *e*_r_: Relative permittivity; *µ*: Dipolar moment; *g*(*e*_r_)-*f*(n): Orientation polarization; dH: Hildebrand parameter.

Solvent	*n*	*e*_r_	*m*	*g*(*e*_r_)-*f*(n)	dH
**Cyclohexane**	1.426	2.024	0	0	16.8
**Ethyl Acetate**	1.372	6.03	1.78	0.41	18.4
**Dichloromethane**	1.424	9.02	1.6	0.46	20.3
**Ethanol**	1.359	24.55	1.66	0.62	26.2

**Table 2:** Weight fraction extracted with each organic solvent evaluated. B3: Coil 3; B4: Coil 4.

Sample	Total Extracted Fraction	Fraction with Ethanol	Fraction with Ethyl Acetate	Fraction with Cyclohexane	Fraction with Dichloromethane
B3	0.219	0.133	0.044	0.016	0.026
B4	0.532	0.361	0.054	0.117	ND

ND: Not detected.

**Table 3:** TG data from the B3 sample and their extracts.

Sample	Comp A	Comp B	Comp C	Carbon	CO_2_ %	CaCO_3_ %	CaO %	% Solvent	% Organic	% Inorganic Fraction	% Inorganic without CaO
7B3M	29.23	20.90	24.21	0.67	3.81	8.66	4.84	3.05	75.01	18.13	13.29
7B3E	80.18	0	0	15.63	0	0	0	2.51	95.81	1.68	1.68
7B3PE	58.98	29.50	0	10.33	0	0	0	0.38	98.81	0.80	0.81
7B3AE	38.65	43.46	0	10.90	0	0	0	6.42	93.01	0.57	0.57
7B3CH	54.19	14.30	18.49	5.98	0	0	0	3.62	93.05	3.33	3.33
7B3DM	87.79	5.234	0	1.78	0	0	0	5.197	94.80	0	0
7DB3	3.63	49.30	0	4.50	8.69	19.78	11.06	3.22	57.42	30.64	19.58

**Table 4:** Mean stickies present in the samples after the enzyme treatment with the enzyme Lipase 30 G and SebOil DG at different concentrations.

**Stickies (Million Particles/mL)**	**Control**	**Lipase 30 G Concentration (g/L)**			
		**0.11**	**0.22**	**0.33**	**0.44**
Media	156.44	130.64	121.94	112.44	100.76
Standard Deviation	14	9	9	14	8
% Removal	-	16.49	22.05	28.12	35.59
**Stickies**	**Control**	**SebOil DG Concentration (g/L)**			
		**0.11**	**0.22**	**0.33**	**0.44**
Media	145.03	124.12	126.02	113.80	118.42
Standard Deviation	10	2	4	9	4
% Removal	-	14.41	13.10	21.5	18.34

**Table 5:** Mean stickies removal for treatments with the commercial enzymes at 0.44 g/L, measuring stickies in paper gravimetrically.

	SebOil DG	Lipase 30 G
**% Stickies without Treatment (Control)**	1.43	2.55
**% Stickies with Enzyme**	1.06	0.63
**% Stickies Removal**	25.87	75.29
